# Weak association between socioeconomic Care Need Index and primary care visits per registered patient in three Swedish regions

**DOI:** 10.1080/02813432.2021.1928836

**Published:** 2021-06-07

**Authors:** Anders Anell, Margareta Dackehag, Lina Maria Ellegård

**Affiliations:** a Department of Business Administration, Lund University, Lund, Sweden; b Department of Economics, Lund University, Lund, Sweden; c Department of Economics, Lund University and Faculty of Business, Kristianstad University, Kristianstad, Sweden

**Keywords:** Family practice, health economy, health services research, quality development, statistics, Care Need Index, risk-adjusted payment

## Abstract

**Objective:**

The objective was to examine the association between primary care consultations and a Care Need Index (CNI) used to compensate Swedish primary care practices for the extra workload associated with patients with low socioeconomic status.

**Design:**

Observational study combining graphical analysis with linear regressions of cross-sectional administrative practice-level data.

**Setting:**

Three Swedish regions, Västra Götaland, Skåne and Östergötland (3.5 million residents). Outcomes were measured in February 2018 and the CNI was computed based on data for 31 December 2017.

**Subjects:**

The unit of analysis was the primary care practice (*n* = 390).

**Main outcome measures:**

i) Number of GP visits per registered patient; ii) Number of nurse visits per registered patient; iii) Number of morbidity-weighted GP visits per registered patient; iv) Number of morbidity-weighted nurse visits per registered patient.

**Results:**

The linear associations between the CNI and GP visits per patient were positive and statistically significant (p<0.01) for both the unweighted and weighted measure in two regions, but the associations were mainly due to 10 practices with very high CNI values. The results for nurse visits varied across regions.

**Conclusions:**

For most levels of the CNI, there was no association with the number of consultations provided. This result may indicate insufficient compensation, weak incentives to spend the money, decisions to spend the money on other things than consultations, or stronger competition for patients among low-CNI practices. The result of this observational study should not be taken as evidence against the possibility that the CNI adjustment of capitation may have affected the socioeconomic equity in GP and nurse visits.

**Key Points**
- Swedish primary care practices receive extra compensation for socioeconomically deprived patients but it is unknown how this affects service provision.
- Practice-level data from three regions years 2017-2018 indicate weak or no relation between the socioeconomic burden and the number of physical consultations per patient.
- Results are similar when adjusting for patients' morbidity levels, suggesting that the weak gradient was not explained by longer consultations.
- The exception is that a small number of practices with very high burdens provide more consultations per patient.
- The results may reflect insufficient compensation, lack of incentives, or funds being spent on other things than consultations.

## Introduction

Although socioeconomic inequalities in health and health care utilization prevail in most societies, primary care tends to stand out as a part of health care that benefits individuals with low socioeconomic status (SES) to a relatively high degree [1–3]. A prerequisite for this positive outcome on equity is that general practitioners (GPs) distribute consultations according to differences in patient needs. In practice, several other factors may influence the distribution of GP consultations, including user fees and health literacy across the population as well as shortages of GPs and incentives related to funding and competition for patients [4].

In Sweden, a series of major primary care reforms implemented a decade ago changed incentives for GPs and primary care practices and spurred concerns that low-SES patients would become more disadvantaged. The reforms, which were rolled out in all 21 regional health care authorities, granted patients free choice of provider and free establishment for providers [5]. In combination with new payment systems letting ‘money follow the patient’, there was a concern that low-SES patients, with generally worse health and health literacy [6], would be less profitable and thus unattractive to providers [7].

One linked concern was that most new establishments would locate in richer areas, thus reinforcing the recruitment problems already experienced by primary care providers in low-SES areas [7,8]. Another concern related to the shift of payment systems, in most regions from block grants combined with fee-for-service (FFS, in this setting visit fees) to capitation (a lump-sum per listed individual). Results from economic experiments [9–11] and observational studies [12–14] indicate that capitation (compared to FFS) reduces the number of services provided, particularly for patients with higher needs. From an international perspective, Swedish GPs see fewer patients per day and nurses provide more primary care [15].

Studies of the Swedish reforms offer mixed support for these concerns. Although new establishments were more likely to locate in affluent areas, the correlation was mostly explained by region-level differences [16,17]. Within the first post-reform years, the distribution of physician consultations remained pro-poor in the three largest regions (Stockholm, Västra Götaland and Skåne) [18], although the number of consultations increased relatively more for high-income individuals in Region Skåne [4].

The limited impact on SES-related equity might reflect the possibility that regional health authorities had foreseen, and tried to prevent, the potentially disadvantageous effects on low-SES patients. Indeed, the tendency of new establishments in affluent areas was significantly dampened in regions where the capitation payment was higher for low-SES patients [16]. As of 2018, all regions had started to weigh the capitation in this way, using the so-called Care Need Index (CNI). The CNI quantifies the perceived workload associated with different patient characteristics (Table 1) [19,20]. The index has been shown to correlate with other measures of social deprivation and with self-reported health in the Stockholm region [21]. In Region Östergötland, primary care costs per patient were positively correlated with socioeconomic deprivation before the reform (in 2006) [22] but negatively, yet insignificantly, correlated with the CNI afterward (in 2013) [23]. Notably, these analyses were carried out before Östergötland started to use the CNI to adjust the capitation.

**Table 1.** Factors increasing the CNI*.

	Relative CNI weight
Being under five years of age	3.23
Being born in Europe outside the European Union, or in Africa, Asia, or South America	5.72
Being over 65 years and living alone	6.15
Being a single parent with children under 17 years	4.19
Being above one year of age and having recently moved across parish borders	4.19
Being 16–64 years and unemployed	5.13
Being 25–64 years and having at most nine years of compulsory schooling.	3.97

*Care Need Index (CNI).

When payment is adjusted by the CNI, primary care providers in low-SES areas receive more income. However, it is not known whether high-CNI providers spend this additional money to provide more or different care for their patients. Providers may use the additional resources in any way they see fit, e.g. recruiting more general practitioners or nurses, but they may also retain the money as surpluses or spend it in ways not benefiting low-SES patients. The primary aim of this study was to examine the relationship between the CNI and consultations per patient in 2018 using data from three regions (Västra Götaland, 1.7 mn residents, Skåne, 1.3 mn residents, Östergötland, 0.5 mn residents). The secondary aim was to analyse if the relationship differed when using a weighted visit measure accounting for patient morbidity. The rationale was the observation that a consultation is more resource-intensive for patients with greater needs; the raw number of visits cannot, whereas the weighted measure might, capture the possibility that the CNI-based payment makes providers allocate more time to sicker patients. If an increase in the CNI is not associated with the raw number of visits but positively associated with the weighted measure, this may indicate that sicker patients benefit from the CNI compensation in the sense that the given number of visits to a larger extent is allocated to the sicker patients.

## Material and methods

### Institutional background

In Sweden, primary care is commonly provided by a mix of public and private multi-professional group practices, staffed by general practitioners, nurses and other disciplines such as physiotherapists, occupational therapists and social workers [5]. These primary care centers (PCC) operate under a standard contract with their regional health authority. Each region has discretion over the required service package and payment principles.

In 2018, the three study regions reimbursed PCCs almost entirely by capitation (a monthly sum per registered patient). The regions used CNI to adjust payment since 2009 (Skåne and Västra Götaland) and 2014 (Östergötland) . In Skåne and Östergötland, 20% and 12% of the base capitation was weighed by the CNI, so that PCCs with above (below) the regional mean CNI received higher (lower) payment. In Västra Götaland, 2% of the primary care budget was redistributed to PCCs with CNI > 2.5, receiving a fixed sum per CNI point above the threshold per listed patient [24–26].

### Study population

The unit of analysis was the PCC. The study population consisted of all PCCs active in our study regions in February^1^ 2018, excluding three units with few registered patients.

### Data

We used data from regional databases on visits at PCCs in 2017–2018 and the list size of each PCC in 2018. The data included information on consultation date, PCC identifier, professional category (GP/nurse), up to 8 ICD10 diagnoses, and PCC enrollment spell dates. CNI was computed using individual background data (as of 31 December 2017) obtained from Statistics Sweden. We studied both GP and nurse consultations.

### Methods

We calculated the CNI of each listed patient using the official weights [24]. Each PCC was assigned a CNI equal to the average CNI of its patients registered on 1 February 2018.

Our first two outcome measures at the PCC level were calculated as the number of GP visits and nurse visits in February 2018, divided by the number of registered patients (in thousands).

Two morbidity-weighed outcome measures were then calculated as follows. A morbidity index for each patient was computed using the Johns Hopkins ACG^®^ System (v.11.2.1) based on diagnoses registered in 2017. The ACG algorithm classifies patients into groups by expected resource utilization.^2^ Each visit made by patient *i* in Feb 2018 was then weighed by (1 + ACG_i), i.e. visits by patients for whom ACG = 0 were assigned a weight of 1 (no. unweighted visits = no. weighted visits), visits by patients for whom ACG = 1 were weighed by 2, etc. The weighted visit measures were then aggregated to the PCC level as above.

The four outcome measures were plotted against CNI. We then estimated linear regressions with heteroscedasticity-robust standard errors. Separate models were estimated for each region. Apart from raw models including only the CNI as an independent variable, we estimated adjusted models with three covariates: the share of elderly (>65 years) among the PCC’s listed patients, a dummy indicating if the PCC was located in the same postal code area as a hospital with an emergency department, and the number of residents per PCC in the postal code area. The latter two variables were meant to capture the availability of substitutes for the PCC’s services (higher availability in urban areas). For Västra Götaland, we also estimated separate models for PCCs with CNI below or above 2.5, acknowledging the threshold for CNI-adjusted payment. Regressions were estimated for all PCCs, and for a sample excluding the ten PCCs with the highest CNI, corresponding to a CNI > 4.7 (5 PCCs in RS and 5 in Västra Götaland).

In sensitivity analyses, we added the squared CNI value to the regression models, used ACG weights based on diagnoses registered in 2018, and used care consumption data for the whole year instead of only February.^3^

## Results

Table 2 shows descriptive statistics for the PCC-level data by region. PCCs in Östergötland had on average the most GP and nurse visits. Skåne and Västra Götaland had a similar number of GP visits but there were fewer nurse visits in Västra Götaland. There was substantial variation between PCCs, within and across regions, in terms of CNI, poverty rates (share of registered patients with disposable income in the lowest decile of the distribution), age distribution and patient morbidity. For instance, CNI ranged between 1.16 and 6.74 and the lowest poverty rate was 2 percent and the highest 59 percent. The CNI and the poverty rate were almost perfectly correlated (rho = 0.92).

**Table 2.** Summary statistics by region.

Outcome variables (monthly)	Skåne					Västra Götaland					Östergötland				
	*N*	Mean	Std. dev.	Min	Max	*N*	Mean	Std. dev.	Min	Max	*N*	Mean	Std. dev.	Min	Max
GP visits/1000 patients	150	102.6	37.3	35.7	373.3	198	95.8	28.7	47.4	233.0	42	73.1	12.2	48.2	106.3
Weighted GP visits/1000 patients	150	275.4	81.3	110.6	727.8	198	274.4	84.4	131.5	622.1	42	197.7	39.2	125.2	279.3
Nurse visits/1000 patients	150	91.1	28.8	21.2	207.2	198	65.8	26.5	3.4	173.5	42	93.9	25.1	43.4	148.2
Weighted nurse visits/1000 patients	150	307.3	119.5	57.4	682.3	198	238.6	105.3	9.4	614.0	42	420.2	132.4	188.2	768.7

PCC characteristics	Skåne					Västra Götaland					Östergötland				
	*N*	Mean	Std. dev.	Min	Max	*N*	Mean	Std. dev.	Min	Max	*N*	Mean	Std. dev.	Min	Max
Registered patients	150	8 787	3 689	1 845	23 808	198	8 474	3 718	1 704	18 896	42	10 836	4 606	3 088	21 470
CNI	150	2.41	0.78	1.37	5.65	198	2.17	0.90	0.80	6.74	42	2.09	0.71	1.16	4.62
Share poor	150	0.10	0.06	0.03	0.40	198	0.10	0.08	0.02	0.59	42	0.10	0.07	0.03	0.35
Share above 65 years	150	0.20	0.05	0.06	0.35	198	0.20	0.06	0.03	0.34	42	0.21	0.05	0.09	0.32
Mortality	150	0.01	0.00	0.00	0.02	198	0.01	0.00	0.00	0.03	42	0.01	0.00	0.00	0.01
ACG (visitors)	150	2.30	0.44	1.01	3.52	198	2.40	0.59	1.16	4.37	42	3.06	0.65	2.21	5.85
Hospital area	150	0.52	0.50	0.00	1.00	198	0.39	0.49	0.00	1.00	42	0.52	0.51	0.00	1.00
Patient density	150	8 659	2 621	3 315	23 808	198	8 474	2 313	3 080	17 289	42	10 836	3 469	3 088	21 242

Descriptive statistics. Outcome variables and ACG based on visits of all patients, not only registered patients. Other variables refer to registered patients. Poor = household disposable income in 10% lowest decile of the regional income distribution. Mortality = number of patients who died in 2018/number of registered patients. ACG of patients visiting the PCC calculated based on diagnoses set in 2017. The hospital area is a dummy indicating if the PCC is located in the same postal code area as a hospital with an emergency department. Patient density is the number of residents in the postal code area divided by the number of PCCs in the area.

**Figure 1.**
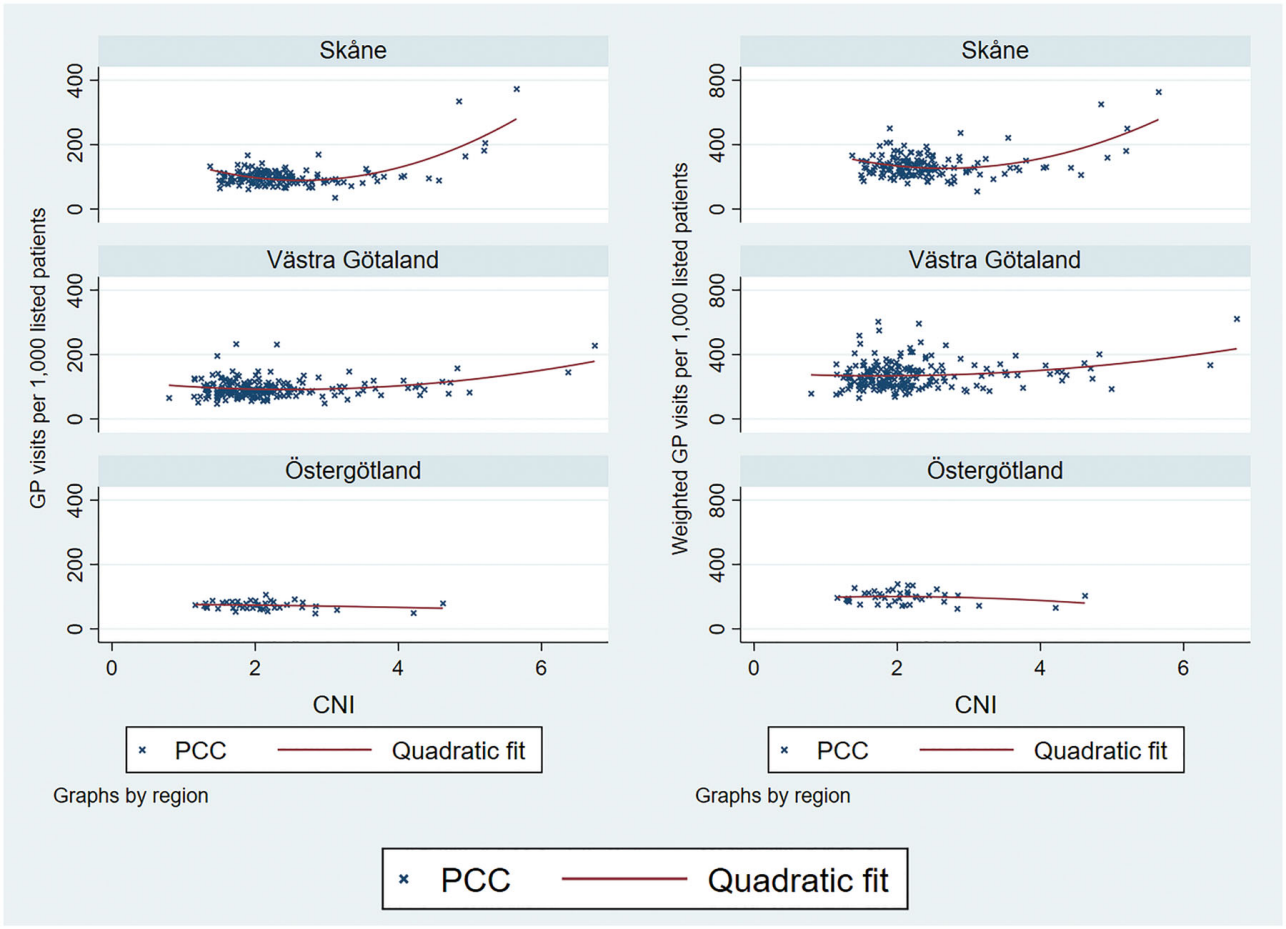
The monthly number of GP visits per 1000 registered patients over Care Need Index values, by region. Left panel: unweighed visits. Right panel: morbidity-weighed visits.

**Figure 2.**
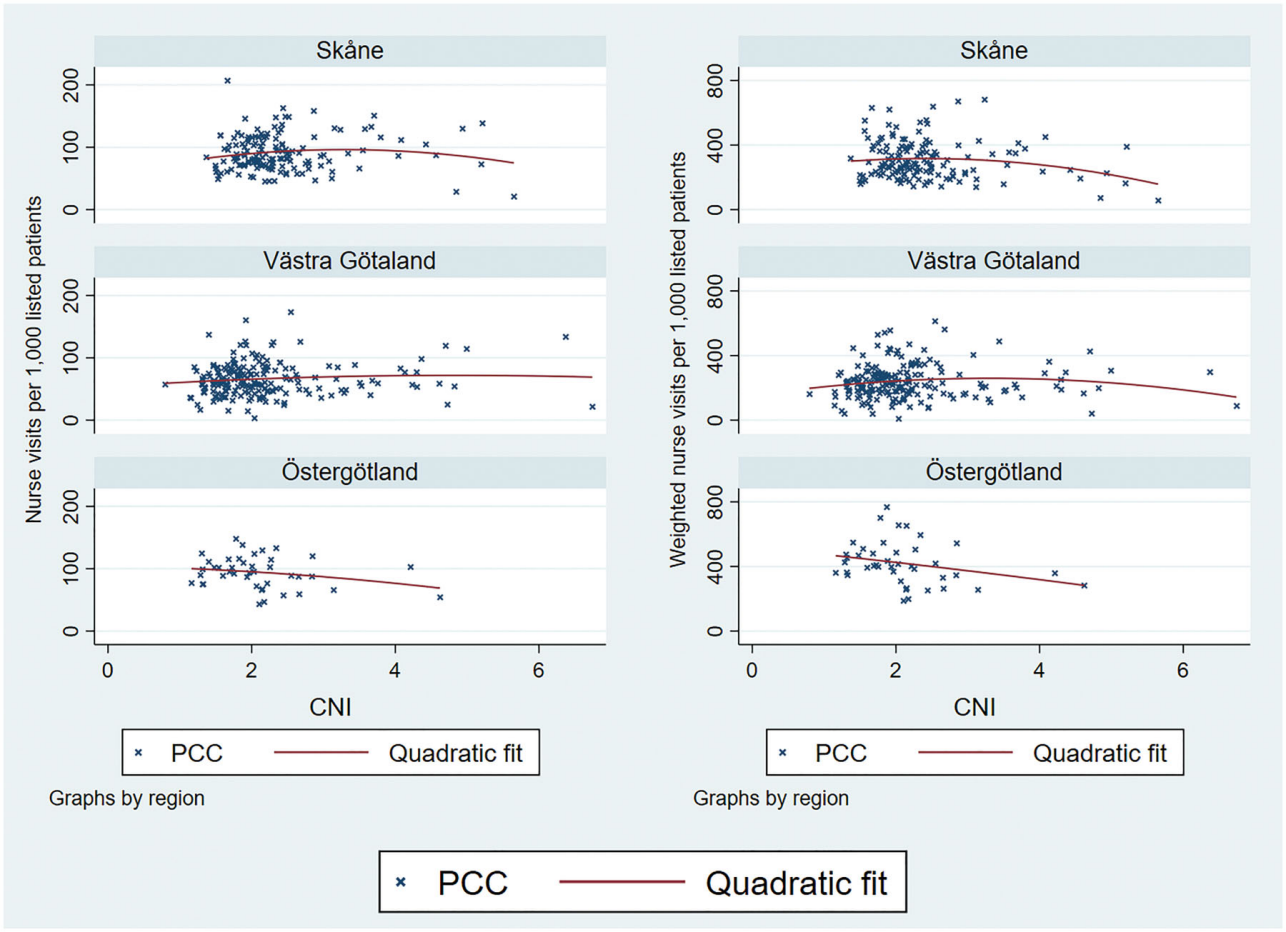
The monthly number of nurse visits per 1000 registered patients over Care Need Index values, by region. Left panel: unweighted visits. Right panel: morbidity-weighted visits.

**Figure 3.**
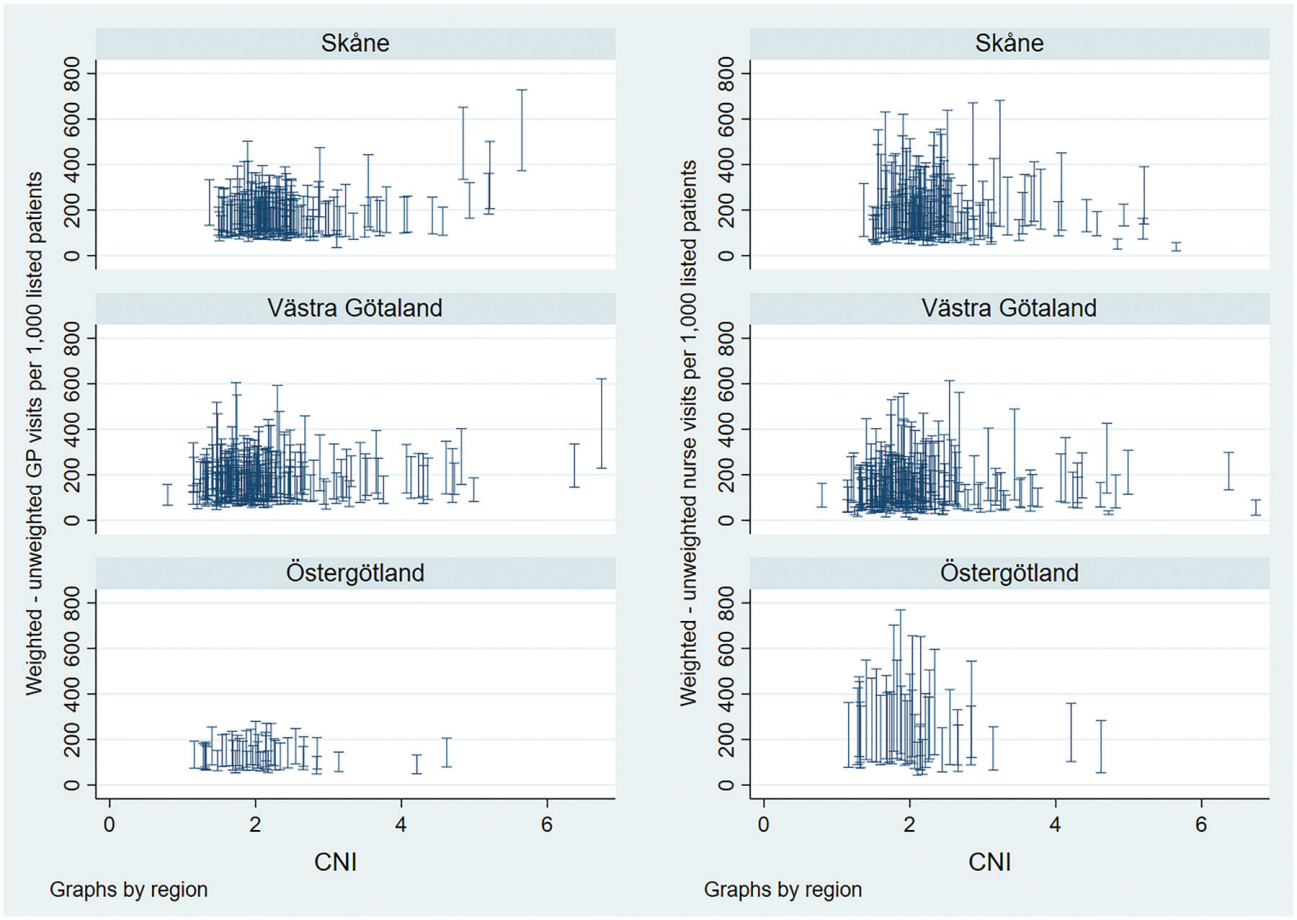
Comparison of weighted and unweighted visit measures, by region. The length of the spikes shows the difference between the weighted and unweighted number of visits per 1000 patients.

The graphical analysis indicated weakly positive associations between GP visits per 1,000 patients and CNI in Skåne and Västra Götaland (Figure 1), due to a small number of PCCs with very high CNI. In Östergötland, the data did not indicate a relationship at any level of the CNI. In no region did the graphs indicate an association between nurse visits and CNI (Figure 2). All patterns were similar for unweighted and morbidity-weighted visit measures, even though the weighting affected PCCs to varying extent (Figure 3).

Table 3 shows the regression estimates. Both the raw and adjusted results showed a positive correlation between CNI and physician visits in Skåne (panel A), but, as expected given the graphical analysis, the result was driven by the five PCCs with the very highest CNI (row ‘Excluding top 5’). A positive correlation between the CNI and nurse visits appeared after removing the top-5-CNI PCCs and adjusting for covariates.

**Table 3.** Regression estimates by region. [–8.739, 11.07]

	Physician visits		Weighted visits		Nurse visits		Weighted visits	
	(1)	(2)	(3)	(4)	(5)	(6)	(7)	(8)
	Raw	Adjusted	Raw	Adjusted	Raw	Adjusted	Raw	Adjusted
Panel A: Skåne								
All	22.11*	27.77**	33.73*	57.00**	0.954	8.617	–22.58	28.69
	[3.819, 40.40]	[8.435, 47.10]	[0.772, 66.69]	[21.85, 92.15]	[–7.510, 9.417]	[–2.021, 19.26]	[–49.31,4.152]	[–6.037, 63.41]
Excluding top 5	–3.605	0.0725	–8.604	9.839	5.699	14.53**	–4.509	47.61*
	[–8.547, 1.336]	[–5.164, 5.310]	[–25.10, 7.893]	[–6.298, 25.98]	[–1.661, 13.06]	[4.042, 25.02]	[–37.71, 28.69]	[6.633, 88.59]
Panel B: Västra Götaland								
All	6.552	10.11**	18.82*	38.03***	2.636	10.70***	3.227	38.56***
	[–0.349, 13.45]	[3.456, 16.76]	[0.958, 36.69]	[20.95, 55.12]	[–2.975, 8.247]	[4.496, 16.91]	[–13.63, 20.08]	[19.91, 57.21]
Only low CNI (<2.5)	0.180	4.364	33.57	46.30*	7.279	12.12*	43.19	58.03**
	[–12.80, 13.16]	[–8.637, 17.37]	[–5.054, 72.19]	[10.15, 82.45]	[–3.983, 18.54]	[2.522, 21.71]	[–0.138, 86.51]	[21.95, 94.12]
Only high CNI (≥2.5)	16.74*	13.41	37.91	57.77*	0.783	12.90	–22.59	38.33
	[2.592, 30.89]	[–4.957, 31.78]	[–4.341, 80.16]	[12.94, 102.6]	[–14.62, 16.19]	[–6.821, 32.63]	[–63.50, 18.32]	[–25.35, 102.0]
Only high CNI, excluding top 5	1.166	0.139	16.27	47.17*	0.386	15.95	–16.66	61.69
		[–14.06, 14.34]	[–14.25, 46.79]	[9.406, 84.94]	[–17.99, 18.76]	[–0.685, 32.59]	[–88.69, 55.36]	[–8.144, 131.5]
Panel C: Östergötland								
All	–3.058	–1.995	–8.394	–1.778	–8.501	0.346	–52.43**	–2.783
	[–9.124, 3.009]	[–10.37, 6.376]	[–25.11, 8.323]	[–24.94, 21.38]	[–18.23, 1.229]	[–10.76, 11.45]	[–83.97, –20.89]	[–39.68, 34.12]

Linear regression estimates of marginal effects of CNI; separate models for each region. In Västra Götaland, low CNI = only PCCs with CNI < 2.5 (no extra payment) and high CNI = only PCCs with CNI> =2.5 (CNI affects payment). Weighted measures weigh each visit with the patient's ACG score. Adjusted models control for share of patients above 65 years, location close to a hospital, and patients per PCC in the postal code area. 95% confidence intervals in brackets. *p<.05, **p<.01, ***p<.001.

For PCCs in Västra Götaland (Table 3, panel B), the CNI was positively correlated with physician visits and, in the adjusted specifications, with nurse visits. The associations were primarily driven by the PCCs with the very highest CNI (row ‘Only high CNI, excluding top 5’), with the interesting exception of a positive correlation with weighted GP visits for low-CNI (<2.5) and high-CNI (≥2.5) PCCs (col. 4). For nurse visits, the associations were significant only for low-CNI PCCs (cols. 6 and 8), although the parameter estimates for low-CNI and high-CNI PCCs were not statistically significantly different from each other.

Panel C shows that the CNI was not significantly correlated with any measure in Östergötland in either raw or adjusted specifications, except for the unadjusted model for weighted nurse visits which indicated a significant negative correlation with CNI.

The sensitivity analysis indicated that the results were stable.

## Discussion

The analysis shows weak associations between CNI payment and primary care consumption. Only the 10 PCCs with the very highest CNI supply more GP visits compared to lower-CNI PCCs. The adjusted analyses also show positive associations between CNI and nurse visits in both Skåne and Västra Götaland. However, the analysis of Västra Götaland revealed such an association also for PCCs with a CNI below the level at which the region starts to compensate for high CNI. This result highlights the possibility that the associations do not reflect reactions to payment. They may for instance reflect a general increase in the demand for primary care at higher CNI levels.

The visit count is not an ideal proxy for resource use, as visit lengths may vary across patients. We could not measure visit length, but the results were similar when we used patient-level morbidity to assign a higher weight to visits made by sicker patients. This result suggests that the weak gradient was not explained by longer consultation lengths in high-CNI PCCs.

As the pattern was similar across regions for the most commonly observed CNI values, regional differences in the way the CNI affected payment did not seem to matter in this part of the CNI range. The contrast between the top-five and other PCCs in Skåne and Västra Götaland – the regions in which the CNI adjustment compensates for high CNI the most – may possibly be a sign that the adjustment only noticeably affects payment for PCCs with very high CNI values.

The shortage of GPs in Swedish primary care is particularly challenging for PCCs in rural and poor areas and may result in more rationing of GP visits than what is common practice. Our results may reflect that the additional resources were insufficient to alleviate the recruitment problem [8]. The results for Skåne suggest that PCCs react differently to increasing CNI depending on their geographical location: The (urban) PCCs with the very highest CNI increase the number of GP visits, whereas PCCs in rural areas, who rely on more on district nurses, provide more nurse consultations.

The results do not rule out that the CNI compensation was used on other dimensions of care, for instance, preventive outreach activities to areas with low health literacy, or on increased telephone or mail contacts which are not included in the visit measures. We may thus underestimate the association between CNI and primary care consumption.

It is also possible that the extra funding was not used at all. A previous descriptive report of public PCCs in Skåne found that higher CNI payment correlated with improved financial results of practices [27]. If funds are not used by providers as intended, it may be appropriate to strengthen the monitoring of activities in combination with the implementation of knowledge support systems regarding e.g. evidence-based outreach preventive services.

Additional explanations behind the weak relationship between CNI and the number of GP or nurse visits per registered patient exist. It is possible that both low and high CNI PCCs provide more consultations but for different reasons – the low-CNI PCC because of more intense competition for patients; the high CNI PCC because of the extra payment. In this case, it would be detrimental for high CNI patients at low CNI PCCs if regional healthcare authorities would reduce the extra payment. Improved monitoring and implementation of knowledge support systems is the favourable policy option.

### Limitations

A fundamental limitation of the analysis is that the models are not causal. The cross-sectional analysis provides a snapshot of the situation in Swedish primary care but does not reveal whether PCCs with high CNI would have provided even fewer visits per patient, had they not received additional funding based on CNI. In particular, the results of our analysis are not evidence against the possibility that the CNI adjustment of capitation has served to limit the impact on socioeconomic equity in GP and nurse visits of the previous choice reforms [16,17].

This analysis studies the question of whether CNI payment at the PCC-level associates with more primary care use and was thus conducted using PCC-level data. However, it is important to recognize that the study does not consider how resources are distributed between low-CNI and high-CNI patients within a PCC. For the CNI payment to improve socioeconomic equity in healthcare and health, the additional resources should be used in ways benefitting the group that the payment system intends to target.

As for the morbidity-weighted measure, the fact that the weights were based on diagnoses set during 2017 implies too low weights for patients with the first onset of disease in early 2018. The results were similar when using morbidity weights from diagnoses set in 2018, though.

As ACG also affects the capitation, PCCs may overreport diagnoses to increase their average ACG and thus boost their payment [28]. If PCCs with high (or low) CNI are more (or less) likely to subsume to this incentive, the correlation between our weighted measure and CNI will be biased. However, the ACG and CNI displayed a very low correlation (rho=–0.09), suggesting that this concern is negligible.

## Conclusions

Swedish healthcare authorities allocate money to PCCs partly based on the Care Need Index to ascertain that the higher care needs of low SES patients are met. This study compared the number of visits per registered patient across CNI levels in three regions. The results did not indicate that high-CNI (i.e. low-SES) PCCs provided more visits to GPs, with the exception of a small number of PCCs with very high index values in two regions, where there was also a positive association with nurse visits. The results were similar when taking patients’ morbidity levels into account. However, the research design does not allow us to rule out that low-SES PCCs would have provided even fewer consultations in the absence of the CNI-adjustment, or that the detected associations are driven by increased care need rather than responses to payment. To advance further, future studies should preferably employ quasi-experimental designs.

## Notes

1 Two PCCs lacked data from January.

2 In Västra Götaland, the ACG was calculated using only diagnoses set in primary care; in the other regions, we also included diagnoses registered in secondary care. The ACGs based on primary care cost weights from 2013 mapped to primary care diagnoses (Västra Götaland) or all diagnoses (other regions).

3 We preferred the model for February because the CNI was calculated based on background characteristics as of 31 Dec 2017 and thus describes the patient population better in early 2018. (January would be even better, but the data was incomplete for two PCCs, see endnote 1.)
